# Association of the Fibrosis‐4 Index With Indices of Atherosclerosis in Patients With Type 2 Diabetes: An Exploratory Subanalysis of a Prospective Observational Cohort Study

**DOI:** 10.1155/jdr/4671246

**Published:** 2026-04-13

**Authors:** Kenichi Tanaka, Yosuke Okada, Tomoya Mita, Keiichi Torimoto, Fumiya Sato, Naoto Katakami, Hidenori Yoshii, Keiko Nishida, Yoshiya Tanaka, Ryota Ishii, Masahiko Gosho, Iichiro Shimomura, Hirotaka Watada

**Affiliations:** ^1^ Department of Rheumatology and Diabetes, Wakamatsu Hospital of the University of Occupational and Environmental Health, Kitakyushu, Japan, uoeh-u.ac.jp; ^2^ First Department of Internal Medicine, School of Medicine, University of Occupational and Environmental Health, Kitakyushu, Japan, uoeh-u.ac.jp; ^3^ Clinical Research Center, Hospital of the University of Occupational and Environmental Health, Kitakyushu, Japan, uoeh-u.ac.jp; ^4^ Department of Metabolism and Endocrinology, Juntendo University Graduate School of Medicine, Tokyo, Japan, juntendo.ac.jp; ^5^ Department of Metabolic Medicine, Osaka University Graduate School of Medicine, Osaka, Japan, osaka-u.ac.jp; ^6^ Nishida Keiko Diabetes Clinic, Kitakyushu, Japan; ^7^ Department of Biostatistics, Institute of Medicine, University of Tsukuba, Ibaraki, Japan, tsukuba.ac.jp

**Keywords:** atherosclerosis, carotid intima-media thickness, continuous glucose monitoring, fibrosis-4 index, metabolic dysfunction-associated steatotic liver disease, type 2 diabetes

## Abstract

**Background:**

Metabolic dysfunction–associated steatotic liver disease (MASLD) is characterized by insulin resistance and metabolic syndrome, with type 2 diabetes mellitus (T2DM) as a major risk factor. Additionally, MASLD has been linked to liver fibrosis, which is associated with increased cardiovascular risk. Although noninvasive liver fibrosis markers are widely used, the association of these markers with atherosclerosis and continuous glucose monitoring (CGM) indices in T2DM remains unclear. We aimed to investigate the associations of the fibrosis‐4 (FIB‐4) index with atherosclerosis and CGM indices in patients with T2DM.

**Methods:**

This study was an exploratory subanalysis of a prospective observational cohort. Among 999 outpatients with T2DM who underwent baseline CGM, 887 patients (excluding heavy alcohol consumers) were analyzed. FIB‐4, a noninvasive marker of liver fibrosis, was calculated. Associations of FIB‐4 with carotid ultrasound, vascular wave velocity tests, and CGM indices were explored.

**Results:**

After adjusting for disease duration, body mass index (BMI), glycated hemoglobin (HbA1c), gamma‐glutamyl transferase, and complications, FIB‐4 severity category showed significant associations with mean intima‐media thickness (IMT), common carotid artery (CCA) maximum IMT, and brachial–ankle pulse wave velocity in ordinal logistic analyses. Consistent with the ordinal logistic analyses, FIB‐4 was independently associated with mean IMT in multivariable linear regression. Among the CGM indices, FIB‐4 was positively associated with both mean glucose and the standard deviation (SD) of mean glucose. Moreover, FIB‐4 was negatively associated with time in range (TIR) and positively with time above range (TAR).

**Conclusions:**

In Japanese patients with T2DM, FIB‐4 is associated with carotid thickening and arterial stiffness. Moreover, FIB‐4 correlates with the duration of hyperglycemia, suggesting that liver fibrosis may be linked to atherosclerosis through mechanisms involving poor glycemic control. Assessment of liver fibrosis in T2DM is imperative, necessitating monitoring of atherosclerosis and cardiovascular diseases (CVDs) in cases of suspected advanced liver fibrosis.

**Trial Registration:**

The study has been registered (UMIN000032325) in the University Hospital Medical Information Network Clinical Trials Registry, a nonprofit organization in Japan that meets the requirements of the International Committee of Medical Journal Editors.

## 1. Introduction

Type 2 diabetes mellitus (T2DM) is strongly associated with insulin resistance, often due to obesity or metabolic syndrome. T2DM increases the risk of not only microvascular complications but also atherosclerotic diseases, such as myocardial infarction and cerebrovascular disorders [1]. Therefore, preventing the progression of atherosclerosis in such patients is crucial for extending healthy life expectancy.

With the increasing prevalence of obesity and fatty liver, the incidence of nonviral liver diseases, particularly nonalcoholic fatty liver disease (NAFLD) and nonalcoholic steatohepatitis (NASH), is rapidly increasing. Recently, NAFLD and NASH have been renamed to metabolic dysfunction–associated steatotic liver disease (MASLD) and metabolic dysfunction‐associated steatohepatitis (MASH), respectively, and redefined [2]. The major underlying features of MASLD/MASH include exacerbated insulin resistance and metabolic syndrome, with T2DM being strongly associated with the development of MASLD/MASH [3]. In this context, metabolic dysfunction, characterized by insulin resistance and related cardiometabolic abnormalities, can be viewed as a common pathophysiological background linking atherosclerosis and MASLD/MASH [4, 5]. In addition, the presence of diabetes is an independent factor associated with liver fibrosis in MASLD/MASH [6]. Progression of liver fibrosis is associated with poor prognosis, including the development of cirrhosis, hepatocellular carcinoma, liver‐related mortality, and increased risk of cardiovascular events, particularly in individuals with advanced fibrosis [7, 8]. Moreover, MASLD has been proposed as a systemic metabolic disorder with cardiovascular complications, supporting the clinical relevance of evaluating liver fibrosis in relation to vascular risk [9].

Progression of liver fibrosis is clinically important. Accordingly, various noninvasive markers and clinically based scoring systems, including the fibrosis‐4 (FIB‐4) index, have been used to assess fibrosis severity [10–12]. Although there are sporadic reports on the association between noninvasive markers of liver fibrosis and atherosclerotic indices in MASLD patients, only a limited number of studies specifically focused on patients with T2DM. One such study reported a positive association between the FIB‐4 index and carotid intima‐media thickness (IMT), but the sample size in that study was small, and the data analysis did not adjust for important confounding factors [13]. Furthermore, other studies found a close association between MASLD onset and progression with impaired hepatic insulin resistance and decreased insulin clearance [14]. To our knowledge, however, the potential impact of liver fibrosis on continuous glucose monitoring (CGM)–derived metrics has not yet been investigated systematically.

CGM provides clinically useful glucose parameters, including mean glucose levels, various glycemic variability indices, such as standard deviation (SD), % coefficient of variation (%CV), and mean amplitude of glycemic excursions (MAGEs), as well as metrics like time in range (TIR), time above range (TAR), and time below range (TBR). In this regard, the Advanced Technologies and Treatments for Diabetes (ATTD) issued an international consensus in 2019 recommending TIR as a standard metric for diabetes management, underscoring its clinical value [15]. In fact, high TIR values have been reported to be significantly associated with reduced risk of both microvascular and macrovascular complications [16, 17].

The aim of the present study was to determine the associations of noninvasive markers of liver fibrosis, including the FIB‐4, with atherosclerosis and various CGM indices in Japanese patients with T2DM.

## 2. Materials and Methods

### 2.1. Participants

This study targeted Japanese patients with T2DM who regularly attended the outpatient diabetes clinics of 34 clinical institutions across Japan (Supporting Information 1: Methods), and details of the study design and inclusion/exclusion criteria have been published previously [18]. The two inclusion criteria were as follows: (1) age ≥ 30 and <80 years; (2) no change in antidiabetic medications (except insulin dosage) for ≥6 months before signing the informed consent form. The exclusion criteria were the following: (1) type 1 or secondary diabetes; (2) history of myocardial infarction, angina pectoris, stroke, cerebral infarction, or atherosclerosis obliterans; (3) current renal dialysis; (4) liver dysfunction rated as worse than mild (aspartate aminotransferase [AST] ≥ 100 IU/L); (5) moderate or severe heart failure (New York Heart Association Stage III or worse); or (6) use of a sensor‐augmented insulin pump. Patients who met the eligibility criteria were invited to participate. From May 2018 to March 2019, we enrolled 1000 outpatients with T2DM who were confirmed to have no significant changes in medications or glycated hemoglobin (HbA1c) and no history of apparent cardiovascular diseases (CVDs). One patient withdrew consent after enrollment. Of the remaining 999 participants, data of 887 were included in the analysis, after excluding 112 who were considered heavy alcohol drinkers (ethanol volume ≥ 30 and ≥20 g/day for men and women, respectively).

### 2.2. Study Design

This study involved exploratory subanalysis of data from an ongoing observational, prospective cohort study on the relationships between glucose fluctuations (detected using CGM) and the incidence of composite cardiovascular events during a 5‐year follow‐up [18].

### 2.3. Study Endpoints

The primary endpoint was the association of FIB‐4 with carotid IMT. The secondary endpoints included the association of FIB‐4 with CGM indices as well as the association of other noninvasive markers of liver fibrosis with carotid IMT.

### 2.4. Biochemical and Clinical Measurements

Fasting blood samples were obtained during the outpatient visits and subsequently analyzed using standard techniques for measurement of AST, alanine aminotransferase (ALT), lipids, creatinine, HbA1c (National Glycohemoglobin Standardization Program), and platelet count. Urinary albumin excretion (UAE) was measured using a latex agglutination assay on a spot urine sample. The estimated glomerular filtration rate (eGFR) was calculated according to the guidelines issued in the Statement of the Japanese Society of Nephrology: eGFR (mL/min/1.73 m^2^) = 194 × serum creatinine^−1.094^ × Age^−0.287^ × 0.739 (if female) [19].

### 2.5. Noninvasive Test of Liver Fibrosis

From the clinical laboratory data, we used the originally reported formulas to calculate FIB‐4:

FIB‐4 = (age [years] × AST [U/L])/([platelets (10^9^/L)] × √ALT [U/L]) [11].

### 2.6. Measurement of Carotid IMT

Carotid ultrasound was performed by specially trained technicians using the same device for each examination in the respective environments [20, 21]. Briefly, the extracranial common carotid artery (CCA), carotid bulb, and internal carotid artery in the neck were scanned bilaterally in transverse projections and at least three different longitudinal projections. The site of greatest thickness and plaque lesions was identified along the arterial walls. IMT represents the distance between two parallel echogenic lines corresponding to the vascular lumen and the adventitial layer. To avoid variability among readers, all scanning images were electronically stored and emailed to the central office (IMT Evaluation Committee, Osaka, Japan), where an experienced reader, blinded to the clinical characteristics of the patients, read them in random order using automatic digital edge detection software (Intimascope, MediCross, Japan) [22] that calculated the average of ~200 IMT in a segment 2‐cm proximal to the dilated portion of the carotid bulb (mean IMT‐CCA); the CCA‐max‐IMT was defined as the higher value on either side.

### 2.7. Measurement of Brachial–Ankle Pulse‐Wave Velocity

Brachial–ankle pulse‐wave velocity (baPWV) was measured at baseline by blinded well‐trained investigators using an automatic waveform analyzer (BP‐203RPE form; Colin Medical Technology, Komaki, Japan), as described in detail previously [23], with the patients lying supine after a 5‐min rest. Occlusion and monitoring cuffs were placed snugly around the appropriate areas on the upper and lower extremities. Using the oscillometric method, the pressure waveforms from the brachial arteries were recorded simultaneously. Patients with an ankle‐brachial index of ≤0.90 were classified as having peripheral artery disease, and their baPWV data were not included in the analysis in this study.

### 2.8. CGM

The FreeStyle Libre Pro CGM (FLP‐CGM) device (Abbott, Japan) was used in this study [18]. No restrictions on daily activities were imposed on the participants, while they wore the FLP‐CGM. Following completion of the 14‐day recording, the CGM‐stored data were downloaded and analyzed for the following CGM parameters: average glucose, SD, %CV, and MAGE; TIR: 3.9–10.0 mmol/L, TAR: >10.0 mmol/L, TAR: >13.9 mmol/L, TBR: <3.9 mmol/L, and TBR: <3.0 mmol/L [15]. Given that the FLP‐CGM is less accurate during the first 24 h after insertion (days 1–2) and the last 4 days of its 14‐day lifetime [24], the FLP‐CGM data presented in this study are those of the middle 8‐day period.

### 2.9. Statistical Analysis

Data are expressed as mean ± SD or median (interquartile range) for continuous variables and as frequency (proportion) for categorical variables. Spearman’s correlation coefficient and linear regression model were used to assess the relationship between two variables. Continuous data were compared using analysis of variance and the Kruskal–Wallis test, whereas categorical data were compared using the chi‐square and Fisher’s exact tests. Based on the FIB‐4, the participants were divided into three FIB‐4 subgroups: those with low FIB‐4: <1.30, medium: 1.30 to <2.67, and high: ≥2.67 [25]. Multivariate linear regression analysis was performed to determine whether noninvasive tests for liver fibrosis were associated with IMT or baPWV. Univariate or multivariate ordinal logistic regression analysis was performed to determine whether the severity indicated by the noninvasive tests was associated with IMT or baPWV. Based on clinical judgment, potential conventional risk factors that are usually evaluated with clinical, biochemical, or metabolic testing were included in the models. For all tests, a *p*‐value < 0.05 was considered statistically significant. All analyses were performed using SAS version 9.4 (SAS Institute, Cary, NC).

## 3. Results

### 3.1. Participant Demographics

**Figure 1:**
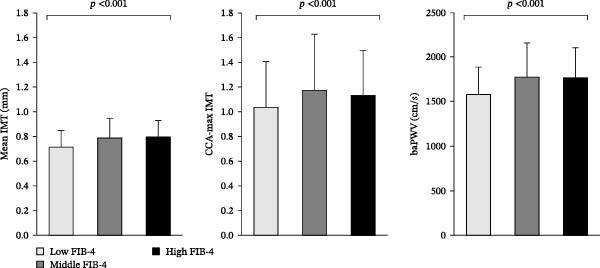
Comparisons of atherosclerosis indices (mean IMT, CCA‐max‐IMT, and baPWV) based on the severity of FIB‐4. Data are mean ± SD. *p*‐Value by the Kruskal–Wallis test. baPWV, brachial–ankle pulse‐wave velocity; CCA, common carotid artery; FIB‐4, fibrosis‐4 index; IMT, intima‐media thickness.

Table 1 presents the baseline characteristics of the study participants (*n* = 887), with a mean estimated duration of diabetes of 13.0 years, HbA1c level of 7.07% (53.7 mmol/L), and FIB‐4 of 1.57. The mean indices of atherosclerosis were an IMT of 0.76 mm, a CCA‐max IMT of 1.11 mm, and a baPWV of 1700.0 cm/s. The mean CGM indices were a glucose level of 7.57 mmol/L, a CV of 26.09%, and a TIR of 79.1%. Table 1 summarizes the FIB‐4‐severity‐stratified comparisons of baseline characteristics of patients with T2DM. The low, middle, and high FIB‐4 groups accounted for 42%, 52%, and 6% of the participants, respectively. Subjects of the low FIB‐4 group were significantly younger and had significantly shorter duration of diabetes, higher body mass index (BMI), higher triglyceride levels, and higher HbA1c levels, although no significant difference was observed in the CGM‐based mean glucose level or other CGM indices. Regarding atherosclerosis indices among the three groups, the mean IMT and CCA‐max IMT were the lowest in the low FIB‐4 group, which also had a lower baPWV (Figure 1). In the analysis of antidiabetic medications, the low FIB‐4 group had higher usage rates of metformin and sodium‐glucose cotransporter 2 inhibitors but lower usage rates of dipeptidyl peptidase‐4 inhibitors.

**Table 1:** Comparison of baseline characteristics of patients with type 2 diabetes by severity of FIB‐4 index.

Variables	All	FIB‐4 index			
		Low (<1.30)	Middle (1.30 to 2.67)	High (≥2.67)	*p*‐Value
Number	887	370	460	57	—
Age (years)	64.7 ± 9.7	58.1 ± 9.7	69.2 ± 6.1	72.4 ± 5.6	<0.001
Male (%)	508 (57.3)	203 (54.9)	273 (59.3)	32 (56.1)	0.418
Estimated duration of diabetes (years)	13.0 ± 8.5	11.2 ± 7.8	14.3 ± 8.8	14.9 ± 9.1	<0.001
Ever smoker (%)	449 (50.7)	207 (55.9)	218 (47.5)	24 (42.1)	<0.001
Alcohol drinking habits (%)	395 (44.8)	167 (45.5)	203 (44.4)	15 (43.9)	0.404
BMI (kg/m^2^)	24.6 ± 3.9	25.5 ± 4.1	24.0 ± 3.6	23.6 ± 3.8	<0.001
Systolic BP (mmHg)	131 ± 15	130 ± 15	132 ± 15	131 ± 18	0.378
Diastolic BP (mmHg)	75 ± 11	77 ± 11	74 ± 10	72 ± 11	<0.001
HbA1c (%)	7.07 ± 0.8	7.14 ± 0.88	7.04 ± 0.71	6.89 ± 0.94	0.015
HbA1c (mmol/mol)	53.7 ± 8.8	54.5 ± 9.6	53.4 ± 7.8	51.8 ± 10.3	0.015
Fasting plasma glucose (mmol/L)	7.57 ± 1.79	7.66 ± 1.88	7.49 ± 1.64	7.65 ± 2.22	0.435
Aspartate aminotransferase (U/L)	23.1 ± 8.7	20.6 ± 6.6	24.0 ± 8.3	32.9 ± 13.4	<0.001
Alanine aminotransferase (U/L)	23.5 ± 13.2	24.5 ± 13.8	22.5 ± 12.7	25.0 ± 12.5	0.005
Gamma‐glutamyl transferase (U/L)	30.2 ± 30.9	28.8 ± 24.5	29.0 ± 31.0	48.3 ± 53.8	0.103
eGFR (mL/min/1.73 m^2^)	72.9 ± 20.9	78.8 ± 22.9	68.1 ± 17.5	73.0 ± 23.1	<0.001
Total cholesterol (mmol/L)	4.77 ± 0.81	4.74 ± 0.81	4.79 ± 0.80	4.76 ± 0.87	0.506
HDL cholesterol (mmol/L)	1.54 ± 0.40	1.49 ± 0.34	1.57 ± 0.41	1.68 ± 0.50	0.003
Triglycerides (mmol/L)	1.34 ± 0.92	1.43 ± 0.93	1.29 ± 0.95	1.17 ± 0.58	0.007
Uric acid (μmol/L)	304.6 ± 71.4	301.8 ± 71.8	308.4 ± 71.6	292.2 ± 65.78	0.188
Urinary albumin excretion (mg/g・creatinine)	103.9 ± 395.1	93.8 ± 337.5	118.0 ± 455.8	54.7 ± 109.0	0.016
Leukocyte count (/μL)	6171 ± 1693	6688 ± 1782	5870 ± 1507	5237 ± 1547	<0.001
Hemoglobin (g/dL)	14.1 ± 1.4	14.3 ± 1.5	14.0 ± 1.4	13.6 ± 1.4	0.007
Platelet count (10^4^/μL)	22.3 ± 5.5	26.1 ± 4.9	20.3 ± 3.8	14.2 ± 3.0	<0.001
FIB‐4 index	1.57 ± 0.77	0.97 ± 0.23	1.81 ± 0.36	3.52 ± 1.11	<0.001
Ultrasonographic scans of the artery (*n*)	536	225	278	33	—
Mean IMT (mm)	0.76 ± 0.15	0.72 ± 0.13	0.79 ± 0.16	0.80 ± 0.14	<0.001
CCA‐max‐IMT (mm)	1.11 ± 0.42	1.03 ± 0.37	1.17 ± 0.45	1.13 ± 0.36	<0.001
Arterial stiffness (*n*)	393	150	216	27	—
baPWV (cm/s)	1700 ± 363	1580 ± 308	1775 ± 381	1767 ± 336	<0.001
CGM metrics					
Mean glucose (mmol/L)	7.57 ± 1.79	7.66 ± 1.88	7.49 ± 1.64	7.65 ± 2.22	0.869
SD (mmol/L)	2.02 ± 0.62	1.98 ± 0.57	2.04 ± 0.64	2.14 ± 0.70	0.252
CV (%)	26.09 ± 5.76	25.55 ± 5.27	26.50 ± 6.15	26.31 ± 5.36	0.078
MAGE (mmol/L)	5.38 ± 1.95	5.27 ± 1.84	5.45 ± 2.00	5.55 ± 2.17	0.370
TIR (%)	79.1 ± 18.5	79.4 ± 19.3	79.3 ± 17.1	74.9 ± 23.7	0.507
TAR^>10 mmol/L^ (%)	18.7 ± 19.1	18.7 ± 19.9	18.2 ± 17.6	23.2 ± 24.8	0.852
TAR^>13.9 mmol/L^ (%)	3.7 ± 9.2	3.6 ± 10.1	3.3 ± 7.2	7.0 ± 15.1	0.411
TBR^<3.9 mmol/L^ (%)	2.2 ± 4.7	1.9 ± 3.8	2.5 ± 5.4	1.9 ± 3.9	0.281
TBR^<3.0 mmol/L^ (%)	0.3 ± 1.6	0.3 ± 1.7	0.4 ± 1.5	0.2 ± 1.0	0.193
Use of medications (%)					
Oral glucose‐lowering agents	796 (89.7)	335 (90.5)	410 (89.1)	51 (89.5)	0.808
Metformin	490 (55.2)	232 (62.7)	237 (51.5)	21 (36.8)	<0.001
Sulfonylureas	112 (12.6)	35 (9.5)	69 (15.0)	8 (14.0)	0.050
Glinides	63 (7.1)	22 (5.9)	39 (8.5)	2 (3.5)	0.239
Dipeptidyl peptidase‐4 inhibitors	521 (58.7)	193 (52.2)	293 (63.7)	35 (61.4)	0.003
Sodium‐glucose cotransporter 2 inhibitors	202 (22.8)	107 (28.9)	83 (18.0)	12 (21.1)	<0.001
Thiazolidinediones	129 (14.5)	48 (13.0)	74 (16.1)	7 (12.3)	0.420
α‐Glucosidase inhibitors	158 (17.8)	69 (18.6)	79 (17.2)	10 (17.5)	0.856
Glucagon‐like peptide‐1 antagonists	70 (7.9)	38 (10.3)	29 (6.3)	3 (5.3)	0.092
Insulin	141 (15.9)	60 (16.2)	69 (15.0)	12 (21.1)	0.446
Antihypertensive agents	430 (48.5)	171 (46.2)	230 (50.0)	29 (50.9)	0.522
Angiotensin‐converting enzyme inhibitors	25 (2.8)	10 (2.7)	12 (2.6)	3 (5.3)	0.462
Angiotensin II receptor blockers	347 (39.1)	142 (38.4)	183 (39.8)	22 (38.6)	0.921
Calcium channel blockers	241 (27.2)	98 (26.5)	128 (27.8)	15 (26.3)	0.912
Antihyperlipidemia agents	545 (61.6)	194 (52.4)	250 (54.6)	29 (50.9)	0.638
Statins	473 (53.4)	194 (52.4)	250 (54.6)	29 (50.9)	0.756
Antiplatelet agents	41 (4.6)	8 (2.2)	27 (5.9)	6 (10.5)	0.003
Retinopathy (%)	77 (20.8)	77 (20.8)	117 (25.4)	12 (21.1)	0.281
Nephropathy (%)	88 (23.8)	88 (23.8)	140 (30.4)	14 (24.6)	0.092
Peripheral neuropathy (%)	92 (24.9)	92 (24.9)	144 (31.3)	21 (36.8)	0.050

*Note:* Data are the mean ± standard deviation or *n* (%). HbA1c, glycated hemoglobin; SD, standard deviation of mean glucose.

Abbreviations: baPWV, brachial–ankle pulse wave velocity; BMI, body mass index; BP, blood pressure; CCA, common carotid artery; CGM, continuous glucose monitoring; CV, coefficient variation; eGFR, estimated glomerular filtration rate; FIB‐4, fibrosis‐4; FIB‐4, fibrosis‐4 index; FPG, fasting plasma glucose; HDL, high‐density lipoprotein; IMT, intima‐media thickness; MAGEs, mean amplitude of glycemic excursions; TAR, time above range; TBR, time below range; TIR, time in range.

### 3.2. Relationship Between Noninvasive Biomarkers for Liver Fibrosis and Arteriosclerosis Indices or CGM Indices in T2DM

Table S1 summarizes the correlation coefficients between the FIB‐4 index and atherosclerosis indices or CGM indices in patients with T2DM. FIB‐4 correlated with atherosclerosis indices (mean IMT, CCA‐max‐IMT, and baPWV).

### 3.3. Association of Arteriosclerosis Indices With FIB‐4 Severity

In the FIB‐4‐severity‐stratified subgroups, ordinal logistic regression analysis performed to examine whether the index was associated with the atherosclerosis indices (Table 2) showed that the mean IMT, CCA‐max‐IMT, and baPWV were crudely associated with FIB‐4 severity and significantly associated with FIB‐4 severity in the following four models adjusted covariates (Table 2): Model 2 adjusted for sex and positive or negative alcohol consumption; Model 3 adjusted for BMI and duration of diabetes, in addition to the variables adjusted in Model 2; Model 4 adjusted for HbA1c, systolic blood pressure (BP), gamma‐glutamyl transferase, total cholesterol, high‐density lipoprotein (HDL) cholesterol, logarithm of triglycerides, serum uric acid, eGFR, and logarithm of UAE, leukocyte count, and hemoglobin level, in addition to the variables adjusted in Model 3; Model 5 adjusted for smoking status, use of antihypertensive agents, use of antihyperlipidemia agents, use of antiplatelet agents, and presence or absence of diabetic retinopathy, in addition to the variables adjusted in Model 4. The odds ratios (95% confidence interval) of mean IMT, CCA‐max‐IMT, and baPWV were 23.652 (5.034–110.937), 1.894 (1.142–3.141), and 1.109 (1.030–1.194), respectively (Model 5 in Table 2). With age included as a covariate, the associations between FIB‐4 and the atherosclerosis indices were attenuated and became nonsignificant (Table S2).

**Table 2:** Association between atherosclerosis metrics and severity of FIB‐4 index.

Variables	Odds ratio (95% CI)	*p*
Mean IMT (1 mm increase)		
Model 1	24.153 (7.273, 80.212)	<0.001
Model 2	23.193 (6.877, 78.224)	<0.001
Model 3	18.691 (5.636, 61.989)	<0.001
Model 4	15.653 (3.508, 69.835)	<0.001
Model 5	23.632 (5.034, 110.937)	<0.001
CCA‐max‐IMT (1 mm increase)		
Model 1	1.974 (1.310, 2.975)	0.001
Model 2	1.945 (1.289, 2.936)	0.002
Model 3	1.983 (1.301, 3.022)	0.001
Model 4	1.645 (1.005, 2.691)	0.048
Model 5	1.894 (1.142, 3.141)	0.013
baPWV (100 cm/s increase)		
Model 1	1.149 (1.084, 1.218)	<0.001
Model 2	1.151 (1.087, 1.220)	<0.001
Model 3	1.130 (1.065, 1.199)	<0.001
Model 4	1.104 (1.029, 1,185)	0.006
Model 5	1.109 (1.030, 1.194)	0.006

*Note:* Model 1: crude. Model 2: adjusted for gender and positive or negative alcohol drinking. Model 3: adjusted for variables in Model 2 plus body mass index and duration of diabetes. Model 4: adjusted for variables in Model 3 plus HbA1c, systolic blood pressure, gamma‐glutamyl transferase, total cholesterol, HDL cholesterol, logarithm of triglycerides, serum uric acid, estimated glomerular filtration rate, logarithm of urinary albumin excretion, leukocyte count, and hemoglobin. Model 5: adjusted for variables in Model 4 plus smoking status (never smoker, previous smoker, or current smoker), use of antihypertensive agents, use of antihyperlipidemia agents, use of antiplatelet agents, and presence of diabetic retinopathy.

Abbreviations: baPWV, brachial–ankle pulse wave velocity; CCA, common carotid artery; CI, confidence interval; FIB‐4, fibrosis‐4; IMT, intima‐media thickness.

Similarly, linear regression analyses were conducted to examine the association of FIB‐4 with various indices of atherosclerosis (Table 3). FIB‐4 was crudely associated with mean IMT, CCA‐max‐IMT, and baPWV and, in Model 5, significantly associated with mean IMT. The CCA‐max‐IMT and baPWV were significantly associated with FIB‐4 in Models 1–3, but no significant association was observed in Models 4–5.

**Table 3:** Multivariate linear regression analysis with FIB‐4 as the dependent variable in patients with type 2 diabetes.

Variables	Regression coefficient, β (95% CI)	*p*
Mean IMT (1 mm increase)		
Model 1	0.901 (0.491, 1.310)	<0.001
Model 2	0.871 (0.455, 1.287)	<0.001
Model 3	0.785 (0.372, 1.199)	<0.001
Model 4	0.695 (0.220, 1.170)	0.004
Model 5	0.776 (0.297, 1.255)	0.002
CCA‐max‐IMT (1 mm increase)		
Model 1	0.163 (0.014, 0.311)	0.032
Model 2	0.155 (0.006, 0.304)	0.041
Model 3	0.153 (0.005, 0.302)	0.043
Model 4	0.077 (−0.082, 0.236)	0.342
Model 5	0.115 (−0.046, 0.277)	0.160
baPWV (100 cm/s increase)		
Model 1	0.039 (0.019, 0.060)	<0.001
Model 2	0.039 (0.019, 0.059)	<0.001
Model 3	0.032 (0.011, 0.053)	0.003
Model 4	0.024 (0.000, 0.048)	0.052
Model 5	0.021 (−0.003, 0.045)	0.089

*Note:* Model 1: crude. Model 2: adjusted for gender and positive or negative alcohol drinking. Model 3: adjusted for variables in Model 2 plus body mass index and duration of diabetes. Model 4: adjusted for variables in Model 3 plus HbA1c, systolic blood pressure, gamma‐glutamyl transferase, total cholesterol, HDL cholesterol, logarithm of triglycerides, serum uric acid, estimated glomerular filtration rate, logarithm of urinary albumin excretion, leukocyte count, and hemoglobin. Model 5: adjusted for variables in Model 4 plus smoking status (never smoker, previous smoker, or current smoker), use of antihypertensive agents, use of antihyperlipidemia agents, use of antiplatelet agents, and presence of diabetic retinopathy.

Abbreviations: baPWV, brachial–ankle pulse wave velocity; CCA, common carotid artery; CI, confidence interval; FIB‐4, fibrosis‐4; IMT, intima‐media thickness.

### 3.4. Association of CGM Indices With FIB‐4

Linear regression analyses showed a crude association between FIB‐4 and glycemic variability indices (e.g., SD and MAGE), while a significant positive association with SD was observed in Model 5 (Table 4). In Model 5, FIB‐4 was positively associated with TAR^>10 mmol/L^ and TAR^>13.9 mmol/L^ but negatively with TIR.

**Table 4:** Association between CGM metrics and FIB‐4 index.

Variables	Regression coefficient, β (95% CI)	*p*
Mean glucose (1 mmol/L) increase)		
Model 1	0.018 (−0.010, 0.046)	0.211
Model 2	0.018 (−0.010, 0.047)	0.213
Model 3	0.019 (−0.009, 0.047)	0.182
Model 4	0.091 (0.052, 0.129)	<0.001
Model 5	0.089 (0.051, 0.128)	<0.001
SD (1 mmol/L increase)		
Model 1	0.092 (0.011, 0.174)	0.027
Model 2	0.092 (0.010, 0.174)	0.028
Model 3	0.049 (−0.034, 0.132)	0.244
Model 4	0.113 (0.024, 0.202)	0.013
Model 5	0.119 (0.029, 0.208)	0.009
CV (1% increase)		
Model 1	0.006 (−0.002, 0.015)	0.149
Model 2	0.006 (−0.002, 0.015)	0.155
Model 3	−0.001 (−0.010, 0.008)	0.904
Model 4	−0.002 (−0.010, 0.007)	0.685
Model 5	−0.001 (−0.009, 0.007)	0.828
MAGE (1 mmol/L increase)		
Model 1	0.026 (0.000, 0.052)	0.049
Model 2	0.025 (−0.000, 0.051)	0.057
Model 3	0.015 (−0.010, 0.041)	0.240
Model 4	0.025 (−0.001, 0.051)	0.064
Model 5	0.025 (−0.001, 0.052)	0.062
TIR (10% increase)		
Model 1	−0.027 (−0.054, 0.001)	0.054
Model 2	−0.027 (−0.054, 0.000)	0.053
Model 3	−0.024 (−0.051, 0.003)	0.087
Model 4	−0.076 (−0.111, −0.041)	<0.001
Model 5	−0.078 (−0.113, −0.043)	<0.001
TAR^>10 mmol/L^ (1% increase)		
Model 1	0.002 (−0.000, 0.005)	0.085
Model 2	0.002 (−0.000, 0.005)	0.081
Model 3	0.002 (−0.000, 0.005)	0.090
Model 4	0.009 (0.005, 0.012)	<0.001
Model 5	0.009 (0.005, 0.012)	<0.001
TAR^>13.9 mmol/L^ (1% increase)		
Model 1	0.007 (0.002, 0.013)	0.012
Model 2	0.007 (0.002, 0.013)	0.012
Model 3	0.007 (0.002, 0.012)	0.011
Model 4	0.014 (0.007, 0.020)	<0.001
Model 5	0.014 (0.008, 0.021)	<0.001
TBR^<3.9 mmol/L^ (1% increase)		
Model 1	0.003 (−0.008, 0.014)	0.574
Model 2	0.003 (−0.008, 0.014)	0.585
Model 3	−0.001 (−0.012, 0.010)	0.864
Model 4	−0.007 (−0.017, 0.003)	0.161
Model 5	−0.006 (−0.016, 0.004)	0.257
TBR^<3.0 mmol/L^ (1% increase)		
Model 1	−0.004 (−0.037, 0.028)	0.788
Model 2	−0.004 (−0.037, 0.028)	0.791
Model 3	−0.013 (−0.045, 0.019)	0.440
Model 4	−0.023 (−0.052, 0.006)	0.120
Model 5	−0.022 (−0.051, 0.007)	0.138

*Note:* Model 1: crude. Model 2: adjusted for gender and positive or negative alcohol drinking. Model 3: adjusted for variables in Model 2 plus body mass index and duration of diabetes. Model 4: adjusted for variables in Model 3 plus HbA1c, systolic blood pressure, gamma‐glutamyl transferase, total cholesterol, HDL cholesterol, logarithm of triglycerides, serum uric acid, estimated glomerular filtration rate, logarithm of urinary albumin excretion, leukocyte count, and hemoglobin. Model 5: adjusted for variables in Model 4 plus smoking status (never smoker, previous smoker, or current smoker), use of antihypertensive agents, use of antihyperlipidemia agents, use of antiplatelet agents, and presence of diabetic retinopathy.

Abbreviations: CGM, continuous glucose monitoring; CI, confidence interval; CV, coefficient variation; FIB‐4, fibrosis‐4; MAGEs, mean amplitude of glycemic excursions; SD, standard deviation; TAR, time above range; TBR, time below range; TIR, time in range.

## 4. Discussion

This study investigated the association of the noninvasive liver fibrosis marker FIB‐4 with atherosclerosis and CGM indices and found that FIB‐4 was associated with carotid thickening and arterial stiffness in Japanese patients with T2DM. The mean IMT, CCA‐max‐IMT, and baPWV increased with increasing severity of the FIB‐4 index. In addition, multivariate linear regression analysis showed a significant association between FIB‐4 and mean IMT. These associations remained significant even after adjustment for confounding factors, including HbA1c, BP, lipid profile, and renal function. Another novelty of our study is the focus on the FIB‐4 index and the CGM metrics, making this study highly valuable clinically in this regard.

The progression of hepatic steatosis and liver fibrosis is thought to increase cardiovascular risk through multiple mechanisms, including insulin resistance, dyslipidemia, chronic inflammation, and vascular dysfunction. Hepatic steatosis exacerbates insulin resistance and promotes dyslipidemia, which is characterized by high levels of triglycerides, low levels of HDL cholesterol, and increased levels of small, dense low‐density lipoprotein (LDL) particles [26, 27]. In patients with advanced liver fibrosis, chronic systemic inflammation is likely to occur due to elevated levels of inflammatory cytokines, such as tumor necrosis factor‐alpha (TNF‐α) and interleukin‐6 (IL‐6). Furthermore, in patients with MASLD or MASH, enhanced platelet activation and increased levels of coagulation and fibrinolysis inhibitory factors (e.g., plasminogen activator inhibitor 1, fibrinogen, and factor VII) may contribute to atherothrombosis [28, 29].

FIB‐4, a noninvasive marker of liver fibrosis, is not only simple and easy to calculate but also a useful tool for evaluating liver fibrosis [11, 12] and facilitates the prediction of liver‐related complications and mortality [30]. Recent studies have indicated that liver fibrosis is associated with CVDs in patients with MASLD [9] and that the FIB‐4 is useful for predicting the onset of CVDs [31]. However, only a few studies have investigated the association of noninvasive liver fibrosis markers and CVDs in diabetic patients. In a previous study using data on diabetic patients extracted from the database of the National Health and Nutrition Examination Survey (NHANES) conducted in the United States from 1999 to 2008, high FIB‐4 scores were associated with increased risks of all‐cause and CVD mortality [32]. In a longitudinal cohort study using data on patients with obesity or T2DM extracted from the United Kingdom Clinical Practice Research Datalink (CPRD) General Practice Online Data (GOLD) database, FIB‐4 was found to be associated with risks of liver events, cardiovascular events, and mortality [33]. Our study of Japanese patients found that FIB‐4 was associated with carotid thickening or arterial stiffness. This finding suggests a possible association between liver fibrosis markers and risk of CVDs and adds support to the results of the abovementioned studies [32, 33]. Since carotid ultrasound and baPWV can be used as prognostic tools for predicting all‐cause and cardiovascular mortality in patients with diabetes [34, 35], attention is warranted to identify the risks of not only liver fibrosis but also advanced arteriosclerosis in patients with high FIB‐4.

To the best of our knowledge, there are only two reports that examined previously the association between FIB‐4 and IMT. Cheng et al. [36] reported greater IMT in patients with MASLD (defined as the presence of fatty liver and at least one of the following three conditions: overweight/obesity, T2DM, and metabolic dysfunction) than in those with NAFLD (defined as the presence of fatty liver alone). Although their study included more than 9000 patients, the authors did not adjust for confounding factors in their analysis. The second report by Watanabe et al. [13] examined Japanese patients with T2DM and demonstrated a positive association between FIB‐4 and IMT. However, their multivariate analysis was adjusted only for BP, eGFR, and HbA1c. The strength of our study was the adjustment for various confounding factors, such as the duration of diabetes, diabetic retinopathy, and urinary microalbumin.

The secondary outcome of this study was the association of FIB‐4 with CGM indices. FIB‐4 was crudely associated with SD and MAGE. In Model 5, FIB‐4 was positively associated with SD but not significantly with MAGE or %CV. These results suggest a limited association of FIB‐4 with glucose fluctuations. In contrast, FIB‐4 was negatively associated with TIR but positively with TAR. In patients with advanced liver fibrosis, the development of hepatic insulin resistance exacerbates postprandial hyperglycemia and consequently contributes to prolonged duration of hyperglycemia [14]. Notably, a recent study has demonstrated that low TIR values are significantly associated with increased carotid IMT in patients with T2DM [17]. Given our findings that high FIB‐4 values are associated with low TIR values, which in turn have been linked to increased IMT, it is possible that the progression of atherosclerosis associated with liver fibrosis is partly mediated through low TIR. To the best of our knowledge, no study has reported the association of FIB‐4 with TIR or TAR.

This study has some limitations. First, it involved only Japanese patients; therefore, it is not clear if the study findings are applicable to European and American patients in whom obesity and insulin resistance are presumably more prevalent. Second, the presence or absence of fatty liver was not confirmed in this study by liver biopsy or abdominal ultrasonography. Accordingly, a definitive diagnosis of MASLD could not be established in the study subjects. Therefore, misclassification cannot be excluded [37]. Third, noninvasive fibrosis indices including FIB‐4 have limited diagnostic performance for advanced fibrosis and can be influenced by age and comorbidity profiles; therefore, misclassification cannot be excluded, and these indices should be interpreted with caution [38, 39]. However, despite these limitations, FIB‐4 was significantly associated with markers of atherosclerosis in patients with T2DM. These findings support the potential utility of FIB‐4 as a noninvasive cardiovascular risk assessment tool in patients with type 2 diabetes. Although age is a factor included in the formula to calculate FIB‐4, the models used in the multivariate analysis in this study were not adjusted for any factors that are included in this formula. Consequently, the impact of age was not excluded (Tables 2 and 3). As a sensitivity analysis, we re‐estimated the ordinal logistic models with age included as an additional covariate, and the associations between FIB‐4 and the atherosclerosis indices were attenuated and became nonsignificant (Table S2). These findings suggest that the contribution of the age component to the observed associations cannot be ignored, and the results should be interpreted with appropriate caution. Reduced diagnostic accuracy of FIB‐4 in younger individuals has been reported [40]; however, this was not directly assessed in our study. Fourth, the chance of false‐positive findings may increase because the multiplicity of testing for the multiple outcomes and statistical models was not adjusted in our analysis. Finally, because this study was based on subanalysis of baseline data, we were unable to determine the causal relationship between FIB‐4 and atherosclerosis. Moreover, the association of changes in FIB‐4 with changes in IMT and other indicators is unknown. Future longitudinal studies integrating hard cardiovascular outcomes, more specific fibrosis assessment (including advanced imaging and validated biomarkers), and detailed CGM metrics are warranted to clarify causality.

## 5. Conclusions

Our study found that FIB‐4 was associated with carotid thickening and arterial stiffness in Japanese patients with T2DM. Among the CGM indices, FIB‐4 was negatively associated with TIR and positively with TAR. Given that reduced TIR has been linked to increased carotid IMT in previous studies, the progression of atherosclerosis associated with liver fibrosis may be partly mediated by impaired glycemic control. Noninvasive liver fibrosis markers could be a useful tool for cardiovascular risk identification and personalized diabetes management interventions in T2DM, especially in patients with suspected advanced liver fibrosis. Prediction of liver fibrosis in patients with T2DM is crucial in order to prevent the progression of atherosclerosis and the development of CVDs. However, because FIB‐4 has inherent limitations and is influenced by age and comorbidity profiles, these findings should be interpreted with caution.

## Author Contributions

Conceptualization: Kenichi Tanaka and Yosuke Okada. Methodology: Kenichi Tanaka and Yosuke Okada. Investigation: Kenichi Tanaka, Yosuke Okada, Tomoya Mita, Keiichi Torimoto, Fumiya Sato, Naoto Katakami, Hidenori Yoshii, Iichiro Shimomura, and Keiko Nishida. Data curation: Kenichi Tanaka, Yosuke Okada, Tomoya Mita, Keiichi Torimoto, Fumiya Sato, Naoto Katakami, Hidenori Yoshii, and Keiko Nishida. Formal analysis: Ryota Ishii and Masahiko Gosho. Writing – original draft: Kenichi Tanaka. Writing – review and editing: Yosuke Okada, Tomoya Mita, Keiichi Torimoto, Yoshiya Tanaka, and Hirotaka Watada.

## Funding

This work was supported by the Japan Agency for Medical Research and Development (AMED) (to Hirotaka Watada) (Grant JP20ek0210105) and the Manpei Suzuki Diabetes Foundation (to Hirotaka Watada).

## Disclosure

All authors have read and approved the final manuscript.

## Ethics Statement

The study protocol was approved by the Institutional Review Board of each participating institution and conducted in accordance with the Declaration of Helsinki and current legal regulations in Japan. The study was registered in the University Hospital Medical Information Network Clinical Trials Registry (UMIN000032325). Written informed consent was obtained from all participants.

## Conflicts of Interest

The authors declare no conflicts of interest.

## Supporting Information

Additional supporting information can be found online in the Supporting Information section.

## Supporting information


**Supporting Information** Methods: List of participating institutions. Table S1: Correlation coefficients between noninvasive tests for liver fibrosis and atherosclerotic index or CGM metrics in patients with type 2 diabetes. Table S2: Association between atherosclerosis metrics and severity of FIB‐4 index (including age as a covariate).

## Data Availability

The data that support the findings of this study are available from the corresponding author upon reasonable request.
