# Supplementary material for: Association of the Fibrosis‐4 Index With Indices of Atherosclerosis in Patients With Type 2 Diabetes: An Exploratory Subanalysis of a Prospective Observational Cohort Study
Source: J Diabetes Res. 2026 Apr 13;2026:4671246. doi: 10.1155/jdr/4671246 (PMC13073046; doi:10.1155/jdr/4671246)
Supplement: Supplementary file 1 — Supporting Information Methods: List of participating institutions. Table S1: Correlation coefficients between noninvasive tests for liver fibrosis and atherosclerotic index or CGM metrics in patients with type 2 diabetes. Table S2: Association between atherosclerosis metrics and severity of FIB‐4 index (including age as a covariate). [file JDR-2026-4671246-s001.docx]

**Supplementary Material**

**Supporting Information 1: Methods: List of participating institutions**

**Ashiya Central Hospital:** Koji Matsushita, Manabu Narisawa

**Aso Clinic:** Katsumi Aso, Yuko Ando, Fumihiko Sato

**Hagiwara Central Hospital:** Emiko Morita, Keiichi Torimoto

**Hayashi Clinic:** Isao Hayashi

**Inokuchi Clinic:** Nobuo Inokuchi

**Japan Community Health Care Organization Osaka Hospital:** Masahiro Hatazaki, Arichika Deguchi, Azusa Shiraki

**Juntendo Tokyo Koto Geriatric Medical Center (Department of Medicine, Diabetology and Endocrinology):** Ayako Kitamura, Eri Tanabe, Hidenori Yoshii, Tomio Onuma, Tomo Nakajima

**Juntendo University Graduate School of Medicine (Department of Metabolism & Endocrinology):** Eisuke Yasunari, Hideyoshi Kaga, Hiroaki Sato, Hirotaka Watada, Kagemi Takeno, Luka Suzuki, Miwa Himuro, Syuhei Aoyama, Takashi Funayama, Takehiro Katahira, Takeshi Miyatsuka, Tomoya Mita, Yuya Nishida

**Juntendo University Nerima Hospital (Department of Medicine, Diabetes and Endocrinology**)**:** Koji Komiya

**Kanda Naika Clinic:** Satoshi Kawashima

**Kansai Rosai Hospital (Diabetes and Endocrinology):** Tsunehiko Yamamoto

**Kihara Diabetes Clinic:** Yasuyuki Kihara

**Kosugi Medical Clinic:** Keisuke Kosugi

**Kawasaki Hospital (Department of Internal Medicine):** Akihito Otsuka, Jun Murai: **Matsuoka Medical Clinic:** Hirofumi Matsuoka

**Misaki Naika Clinic:** Nobuichi Kuribayashi

**Japan Labour Health and Safety Organization Kyushu Rosai Hospital, Moji Medical Center (Department of Internal Medicine):** Tadashi Arao, Kei Sugai

**Musashino Family Clinic:** Yuichi Kojima

**Nakakinen Clinic:** Maiko Nakata, Miyoko Saito, Takeshi Osonoi, Yusuke Osonoi

**Nakama Municipal Hospital:** Kohei Uriu, Yoshifumi Inada, Kanako Suzuka, Ichiro Takagi

**National Hospital Organization Osaka National Hospital (Diabetes Center):** Ken Kato

**Nishida Keiko Diabetes Clinic:** Keiko Nishida, Akira Kurozumi, Fumi Uemura, Keiichi Torimoto, Maiko Hajime, Manabu Narisawa, Satomi Sonoda, Kumiko Tidiwa

**Osaka General Medical Center (Department of Diabetes and Endocrinology):** Yohei Fujita, Sayoko Shimizu, Masahisa Hata, Yutaka Umayahara

**Osaka Police Hospital (Department of Endocrinology and Metabolism**)**:** Tetsuyuki Yasuda

**Osaka Rosai Hospital:** Kayoko Ryomoto

**Osaka University Graduate School of Medicine (Department of Metabolic Medicine):** Iichiro Shimomura, Naoto Katakami, Takaaki Matsuoka, Mitsuyoshi Takahara, Kazuyuki Miyashita, Hiroyo Ninomiya, Naohiro Taya

**Sasaki Hospital:** Shinichiro Mine, Kenji Koikawa

**School of Medicine, University of Occupational and Environmental Health, Japan (First Department of Internal Medicine):** Yosuke Okada, Akira Kurozumi, Manabu Narisawa, Maiko Hajime, Fumi Uemura, Satomi Sonoda, Kenichi Tanaka, Takashi Otsuka, Kenji Koikawa, Megumi Miyazaki, Akemi Tokutsu, Momoko Habu, Momo Saito,

**Secomedic Hospital:** Satomi Wakasugi, Tomoya Mita

**Shiraiwa Medical Clinic:** Toshihiko Shiraiwa

**Taneda Clinic:** Yoshinobu Taneda

**Takaishi Naika Ichoka Clinic:** Tomoya Mita

**Tobata General Hospital (Department of Internal Medicine):** Kazuko Kanda

**Wakamatsu Hospital of the University of Occupational and Environmental Health:** Keiichi Torimoto, Kosuke Nishio

Supporting Information 2: Table S1. Correlation coefficients between FIB-4 index and atherosclerotic index or CGM metrics in patients with type 2 diabetes

|  | FIB-4 index | |
| --- | --- | --- |
| Variables | r | 95% CI |
| Mean IMT | 0.287 | 0.207, 0.363 |
| CCA-max-IMT | 0.232 | 0.150, 0.310 |
| baPWV | 0.323 | 0.231, 0.409 |
| Mean glucose | -0.015 | -0.081, 0.051 |
| SD | 0.029 | -0.037, 0.095 |
| CV | 0.071 | 0.006, 0.137 |
| MAGE | 0.033 | -0.033, 0.098 |
| TIR | -0.021 | -0.086, 0.045 |
| TAR^>10 mmol/l^ | -0.007 | -0.073, 0.059 |
| TAR^>13.9 mmol/l^ | 0.020 | -0.046, 0.086 |
| TBR^<3.9 mmol/l^ | 0.035 | -0.031, 0.101 |
| TBR^<3.0 mmol/l^ | 0.041 | -0.025, 0.106 |

Abbreviations: CGM, continuous glucose monitoring; FIB-4, Fibrosis-4; CI, confidence interval; IMT, intima-media thickness; CCA, common carotid artery; baPWV, brachial-ankle pulse wave velocity; SD, standard deviation; CV, coefficient variation; MAGE, mean amplitude of glycemic excursions; TIR, time in range; TAR, time above range; TBR, time below range

Supporting Information 3: Table S2. Association between atherosclerosis metrics and severity of FIB-4 index (including age as a covariate)

| Variables | Odds ratio (95% CI) | *P* |
| --- | --- | --- |
| Mean IMT (1 mm increase) |  |  |
| Model 1 | 24.153 (7.273, 80.212) | <0.001 |
| Model 2 | 2.337 (0.600, 9.102) | 0.221 |
| Model 3 | 2.349 (0.603, 9.148) | 0.218 |
| Model 4 | 1.928 (0.344, 10.791) | 0.455 |
| Model 5 | 3.147 (0.530, 18.695) | 0.207 |
| CCA-max-IMT (1 mm increase) |  |  |
| Model 1 | 1.974 (1.310, 2.975) | 0.001 |
| Model 2 | 1.020 (0.643, 1.618) | 0.933 |
| Model 3 | 1.027 (0.642, 1.643) | 0.912 |
| Model 4 | 0.904 (0.520, 1.573) | 0.722 |
| Model 5 | 1.067 (0.604, 1.886) | 0.823 |
| baPWV (100 cm/s increase) |  |  |
| Model 1 | 1.149 (1.084, 1.218) | <0.001 |
| Model 2 | 0.964 (0.899, 1.034) | 0.307 |
| Model 3 | 0.969 (0.903, 1.040) | 0.382 |
| Model 4 | 0.959 (0.882, 1.042) | 0.320 |
| Model 5 | 0.968 (0.888, 1.055) | 0.463 |

Model 1: crude. Model 2: adjusted for age, gender, and positive or negative alcohol drinking. Model 3: adjusted for variables in Model 2 plus body mass index, and duration of diabetes. Model 4: adjusted for variables in Model 3 plus HbA1c, systolic blood pressure, gamma-glutamyl transferase, total cholesterol, HDL cholesterol, logarithm of triglycerides, serum uric acid, estimated glomerular filtration rate, logarithm of urinary albumin excretion, leukocyte count, and hemoglobin. Model 5: adjusted for variables in Model 4 plus smoking status (never smoker, previous smoker, or current smoker), use of anti-hypertensive agents, use of anti-hyperlipidemia agents, use of anti-platelet agents, and presence of diabetic retinopathy.

FIB-4, Fibrosis-4; CI, confidence interval; IMT, intima-media thickness; CCA, common carotid artery; baPWV, brachial-ankle pulse wave velocity
